# The impact of patient, intervention, comparison, outcome (PICO) as a search strategy tool on literature search quality: a systematic review

**DOI:** 10.5195/jmla.2018.345

**Published:** 2018-10-01

**Authors:** Mette Brandt Eriksen, Tove Faber Frandsen

**Affiliations:** University Library, University of Southern Denmark, Campusvej 55, 5230 Odense M, Denmark; Department of Design and Communication, University of Southern Denmark, Universitetsparken 1, DK-6000 Kolding, Denmark

## Abstract

**Objective:**

This review aimed to determine if the use of the patient, intervention, comparison, outcome (PICO) model as a search strategy tool affects the quality of a literature search.

**Methods:**

A comprehensive literature search was conducted in PubMed, Embase, CINAHL, PsycINFO, Cochrane Library, Web of Science, Library and Information Science Abstracts (LISA), Scopus, and the National Library of Medicine (NLM) catalog up until January 9, 2017. Reference lists were scrutinized, and citation searches were performed on the included studies. The primary outcome was the quality of literature searches and the secondary outcome was time spent on the literature search when the PICO model was used as a search strategy tool, compared to the use of another conceptualizing tool or unguided searching.

**Results:**

A total of 2,163 records were identified, and after removal of duplicates and initial screening, 22 full-text articles were assessed. Of these, 19 studies were excluded and 3 studies were included, data were extracted, risk of bias was assessed, and a qualitative analysis was conducted. The included studies compared PICO to the PIC truncation or links to related articles in PubMed, PICOS, and sample, phenomenon of interest, design, evaluation, research type (SPIDER). One study compared PICO to unguided searching. Due to differences in intervention, no quantitative analysis was performed.

**Conclusions:**

Only few studies exist that assess the effect of the PICO model vis-a-vis other available models or even vis-a-vis the use of no model. Before implications for current practice can be drawn, well-designed studies are needed to evaluate the role of the tool used to devise a search strategy.

## INTRODUCTION

The development of systematic reviews is considered a means of enabling clinicians to use evidence-based medicine (EBM) [1], and the number of systematic reviews is growing quickly [2]. As literature searching forms the underlying basis of systematic reviews, the quality of the literature search is crucially important to the overall quality of the systematic review [3]. Although new techniques can automate the process of systematic reviews, such as using text mining to develop search strategies [4], the task of devising the search strategy still requires intellectual contributions from reviewers. In particular, as the search strategy builds upon the review question, formulating the review question is critical to developing the search strategy.

In their 1992 publication in the *Journal of the American Medical Association,* the Evidence-Based Medicine Working Group emphasized the precise definition of the patient problem, the required information needed to resolve the problem, and the ability to conduct an efficient search as the skills required for practicing EBM [5]. In addition to these skills, the use of conceptualizing models to structure a clinical question was introduced in 1995, when Richardson et al. proposed the use of a four-part model to facilitate searching for a precise answer [6]. They stated that a clinical question must be focused and well articulated for all four parts of its “anatomy”: the patient or problem (P); the intervention or exposure (I); the comparison intervention or exposure (C), if relevant; and the clinical outcome of interest (O).

Despite the existence of other models—such as sample, phenomenon of interest, design, evaluation, research type (SPIDER) [7] and setting, perspective, intervention, comparison, evaluation (SPICE) [8]—the PICO model is by far the most widely used model for formulating clinical questions. The purpose of using PICO is considered to be three-fold [9]. First, it forces the questioner to focus on what the patient or client believes to be the single most important issue and outcome. Second, it facilitates the next step in the process—the computerized search—by prompting the questioner to select language or key terms to be used in the search. Third, it directs the questioner to clearly identify the problem, intervention, and outcomes related to specific care provided to a patient.

The PICO model is also frequently used as a tool for structuring clinical research questions in connection with evidence syntheses (e.g., systematic reviews). The *Cochrane Handbook for Systematic Reviews of Interventions* specifies using PICO as a model for developing a review question, thus ensuring that the relevant components of the question are well defined [10]. The PICO framework is primarily centered on therapy questions, and although it can be adapted to formulate research questions related to prognosis or diagnosis, it is less suitable for other types of clinical information needs [11].

In addition to acting as a conceptualizing tool for asking clinical and research questions, the PICO model can be used as a tool for developing search strategies. According to Considine et al., “the PICO Framework should also be used to develop the search terms that are informed by the PICO question, Medical Subject Headings (MeSH) and any other terms deemed to be relevant” [12]. For a default search, the *Cochrane Handbook* suggests employing only search terms for patients, the intervention, and the study type [13], thus reducing the PICO model to P, I, and S/T (i.e., study type or types of study). Alternatively, instead of study type or types of study, the truncated PIC approach emphasizes the comparison intervention or exposure.

Although conceptualizing models are widely used by information specialists, little is known about the impact of using them as tools for developing search strategies. Therefore, the aim of this systematic review was to determine whether the use of the PICO model as a search strategy tool improves the quality of literature searches.

## METHODS

This systematic review was conducted and reported according to quality standards described in the AMSTAR measurement tool [14] and the PRISMA 2009 checklist [15]. Two reviewers independently carried out study selection, evaluation, and data extraction. We resolved discrepancies in our reviews by consensus. Covidence systematic review software (Veritas Health Innovation, Melbourne, Australia) was used to screen, select, and extract data from included studies. The review protocol was registered in the PROSPERO database (CRD42017055217).

### Search strategy

We searched PubMed (Table 1), Embase, CINAHL, PsycINFO, the Cochrane Library, Web of Science, Library and Information Science Abstracts (LISA), Scopus, and the National Library of Medicine catalog on January 9, 2017. After testing and validating our PubMed search strategy using the capture-recapture technique as well as evaluating retrieval of known items [16], we translated the search strategy for use in other databases, adjusting the controlled vocabulary as applicable (supplementary Appendix A). We also examined reference lists and performed citation searching (Web of Science, v.5.23.2, up to February 1, 2017) of included studies to identify other potentially relevant studies.

**Table 1:** PubMed search strategy

Search strategy	
#1	“databases, bibliographic”[MeSH Terms] OR “Computer Literacy” [MeSH] OR “Data mining” [MeSH] OR “Evidence Based Dentistry” [MeSH] OR “Evidence-Based Emergency Medicine” [MeSH] OR “Evidence-based Medicine” [MeSH] OR “Evidence-based Nursing” [MeSH] OR “Evidence Based Practice” [MeSH] OR “Health literacy” [MeSH] OR “Information literacy” [MeSH] OR “literature based discovery” [MeSH] OR “information seeking behavior” [MeSH] “information storage and retrieval” [MeSH] OR “data mining” [MeSH] OR Bibliographic database search [All Fields] OR Bibliographic database searches [All Fields] OR Bibliographic database searching [All Fields] OR Bibliographic databases search [All Fields] OR Bibliographic databases searches [All Fields] OR Bibliographic databases searching [All Fields] OR Computer literacies [All Fields] OR Computer Literacy [All Fields] OR Computerized Literature Searching [All Fields] OR Data file [All Fields] OR Data files [All Fields] OR Data linkage [All Fields] OR Data mining [All Fields] OR Data retrieval [All Fields] OR Data retrieving [All Fields] OR Data source [All Fields] OR Data sources [All Fields] OR Data storage [All Fields] OR Datamining [All Fields] OR Evidence Based Dental Practice [All Fields] OR Evidence Based Dentistries [All Fields] OR Evidence Based Dentistry [All Fields] OR Evidence Based Emergency Medicine [All Fields] OR Evidence based emergency medicines [All Fields] OR Evidence based health care [All Fields] OR Evidence Based Healthcare [All Fields] OR Evidence based healthcares [All Fields] OR Evidence Based Medical Practice [All Fields] OR Evidence Based Medicine [All Fields] OR Evidence Based Nursing [All Fields] OR Evidence Based Practice [All Fields] OR Evidence based professional practice [All Fields] OR Health literacies [All Fields] OR Health literacy [All Fields] OR Information extraction [All Fields] OR Information extractions [All Fields] OR Information literacies [All Fields] OR Information literacy [All Fields] OR Information processing [All Fields] OR Information retrieval [All Fields] OR Information retrieving [All Fields] OR Information seeking behavior [All Fields] OR Information storage [All Fields] OR literature based discovery [All Fields] OR literature retrieval [All Fields] OR Literature retrieving [All Fields] OR Literature search [All Fields] OR Literature searches [All Fields] OR Literature Searching [All Fields] OR Machine readable data file [All Fields] OR Machine readable data files [All Fields] OR Online database search [All Fields] OR Online database searches [All Fields] OR Online database searching [All Fields] OR Online databases search [All Fields] OR Online databases searches [All Fields] OR Online databases searching [All Fields] OR Research Based Medical Practice [All Fields] OR Research Based Nursing Practice [All Fields] OR Research Based Occupational Therapy Practice [All Fields] OR Research Based Physical Therapy Practice [All Fields] OR Research Based Professional Practice [All Fields] OR Review Literature as Topic [All Fields] OR Search strategies [All Fields] OR Search strategy [All Fields] OR State of the art review [All Fields] OR State of the art reviews [All Fields] OR Systematic review topic [All Fields] OR Text mining [All Fields] OR Theory Based Nursing Practice [All Fields]
#2	Pico [All Fields] OR patient intervention comparison outcome [All Fields] OR patient intervention comparator outcome [All Fields] OR (population intervention comparison outcome [All Fields] OR population intervention comparison outcomes [All Fields]) OR problem intervention comparison outcome [All Fields]
#3	#1 AND #2

### Inclusion and exclusion criteria

We considered all primary studies, regardless of design, as eligible for inclusion if they examined PICO as a tool for developing a search strategy (distinct from other methods for developing a search strategy) for identifying potentially relevant studies in any topic area. We excluded review articles but examined their reference lists to identify other potentially relevant studies. We applied no other restrictions, such as those related to languages or publication years, in this review.

### Outcome measures

Our primary outcome measure was the quality of literature searches using two measures: precision and sensitivity [17]. The Cochrane Handbook defines sensitivity as the number of relevant reports found divided by the total number of relevant reports in existence and precision as the number of relevant reports found divided by the total number of reports identified [10]. Our secondary outcome measure was time spent on the literature search.

### Data extraction

We noted and summarized information pertaining to author, year of publication, study design, searchers, search strategy tools, and calculation of sensitivity and precision. Studies that did not evaluate and quantify the quality of the literature searches in terms of both precision and sensitivity were excluded from analysis. Empirical studies show that recall and precision are inversely related. High recall can easily be obtained but will, however, be at the expense of precision. Because a trade-off between recall and precision is unavoidable, one should only evaluate searches with both of these measures [18].

### Risk of bias assessment

No validated criteria exist for assessing the risk of bias in studies evaluating the effect of PICO as a tool for developing the search strategy in terms of the quality of the searches. Therefore, we used a self-developed set of three criteria: (i) searcher skills, (ii) match between model and question, and (iii) performed searches (Table 2). Each criterion consisted of a set of individual considerations and was assessed using the categories “low risk of bias,” “high risk of bias,” and “unclear risk of bias.” If one of the considerations in a criterion was judged as “high risk of bias” or “unclear risk of bias,” the overall judgment for that criterion was “high risk of bias” or “unclear risk of bias,” respectively. We developed the three criteria by consensus; however, this tool was not validated.

**Table 2:** Risk-of-bias criteria

Criterion	Support for judgment	Review authors’ judgment
Searcher skills	Describe the skills of the searchers as well as their prior knowledge in the specific fields of the searched topics.	Searcher skills had bias due to inadequate random allocation of searchers to topics or order of search strategies applied as well as lack of concealment of searcher identity to reviewers.
Fit between model and topic	Describe the chosen models, the topics to which they are applied, and the number of resulting search blocks. Describe how relevance of search results to topic is determined.	Fit between model and topic bias due to inadequate application of models to topics, varying number of search blocks, and relevance assessment not based on a gold standard.
Quality of searches	Describe how the searches are performed and adapted for each database.	Searches performed had bias due to inadequate adaption of searches to each database as well as lack of consistency in search quality across search strategy tools.

#### (i) Searcher skills

The searchers (i.e., study participants or authors) were the individuals performing the literature searches. If the searchers differed in their searching skills, this might have affected the overall results of the study. Thus, if some searchers had more training in literature searching than others, this could introduce a risk of bias. Similarly, if some of the searchers were familiar with the search strategy tools prior to the study, this also increases the risk of bias. Furthermore, if searchers used all included models in the study (e.g., were instructed to use particular conceptualizing models or unguided searching), the order in which the search strategy tools were applied might have affected search behavior, thus, introducing a risk of bias. Finally, although blinding of the searchers is not possible, blinding of the reviewers evaluating the search results is possible and serves to reduce the risk of bias resulting from knowing the identity of the searchers or search strategy tools that were applied.

#### (ii) Match between model and question

Our risk-of-bias assessment for this criterion was based on the consideration that particular conceptualizing models might be developed to fit different topics or quantitative versus qualitative research and might apply to some topics or research areas better than others, which could influence the study results. Recent recommendations show that different review types require different question formats (i.e., different conceptualizing models and, thus, different search strategy tools) [19]. The fit between model and topic cannot be manipulated (e.g., if a research question does not include an intervention, all elements of the PICO model will not be applicable and, thus, will not fit that particular research question). We considered applying a conceptualizing model that was not fit for that particular research area a high risk of bias.

Another aspect of the fit between model and question is the relevance of the obtained search results. As sensitivity and precision measures are based on relevance, the search results need to be assessed for their relevance. Determination of the relevance of the obtained search results is performed ideally using a predefined set of publications (i.e., a gold standard), such as those retrieved in a systematic review, that can serve to assess the relevance of the search results. Alternatively, an expert group could assess the relevance of the retrieved results. A system’s view of relevance (i.e., the ranking of results or a study being present in the search results) is not sufficient [20]. We considered applying precision and recall without considering relevance based on a gold standard or an expert group a high risk of bias.

Finally, the number of search elements or search blocks needs to be considered, regardless of whether the search was unguided or structured by the use of a search strategy tool. All other things being equal, the number of retrieved articles will decrease as the number of blocks is increased. Consequently, the more elements, the fewer hits, which would affect the results of the study in terms of comparing applied search strategy tools. We considered search strategy tools (i.e., conceptualizing model or unguided search) that had a different number of search elements or search blocks a high risk of bias.

#### (iii) Quality of searches

Our risk-of-bias assessment for this criterion was based on our consideration that the quality of the literature searches might impact the results of the study. Searches could be consistently high quality or consistently low quality, which does not in itself imply high risk of bias. However, if the quality of the searches is not consistently high or low, bias can occur. The quality of searches in this case was determined using criteria outlined in the PRESS statement [3], stressing that the criteria and methods depended on the specific databases. If the literature search was not conducted uniformly or if subject headings were not correctly adapted for each database, we considered it to have a high risk of bias.

Due to differences in the comparisons among search strategy tools in the included studies, we did not perform quantitative analyses. We, therefore, did not follow the sections in the PRISMA 2009 checklist [15] that relate to meta-analysis.

## RESULTS

**Figure 1:**
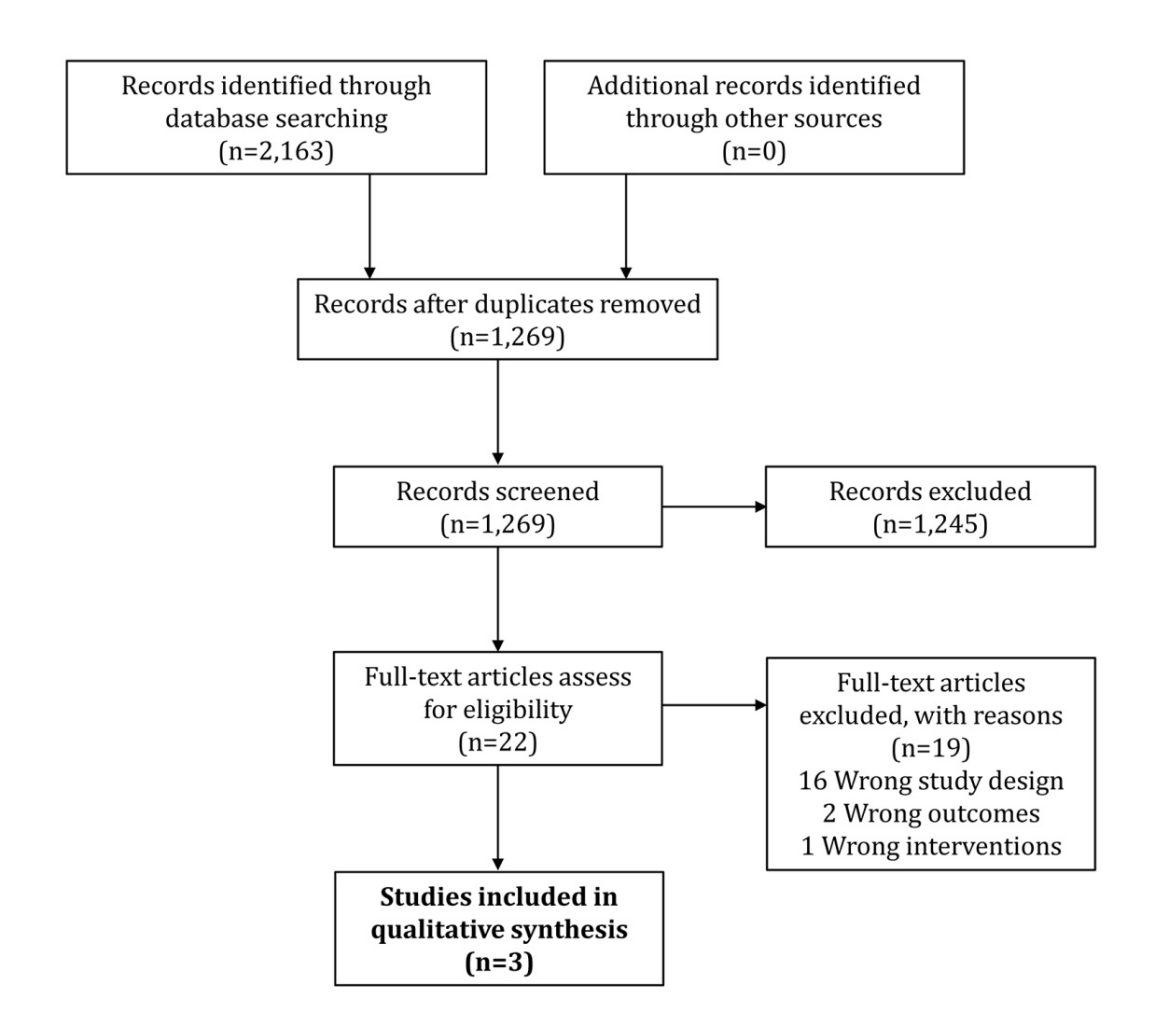
Study selection flow diagram

The literature search identified a total of 1,269 unique records (Figure 1). We assessed 22 full-text articles for eligibility and excluded 19 due to wrong study design (i.e., studies that did not examine PICO as a tool for developing a search strategy for identifying potentially relevant studies in any topic area), wrong outcomes, or wrong interventions (supplementary Appendix B). Therefore, three studies were included in the qualitative analysis [21–23] (Table 3).

**Table 3:**
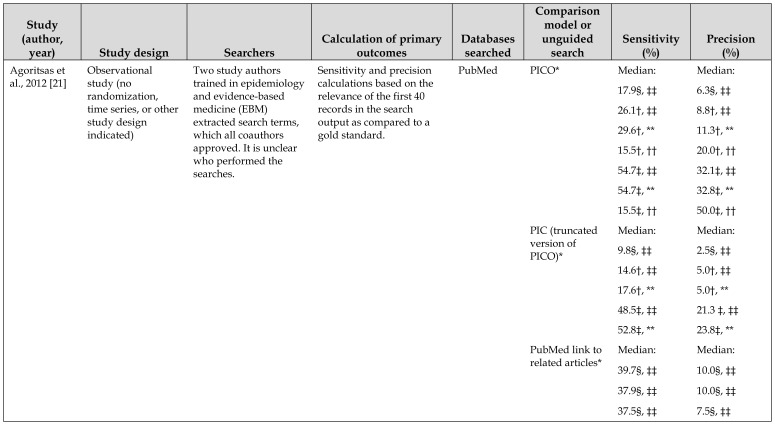
Use of the patient, intervention, comparison, outcome (PICO) model compared to another conceptualizing model as a literature search strategy tool

Study (author, year)	Study design	Searchers	Calculation of primary outcomes	Databases searched	Comparison model or unguided search	Sensitivity (%)	Precision (%)
Agoritsas et al., 2012 [21]	Observational study (no randomization, time series, or other study design indicated)	Two study authors trained in epidemiology and evidence-based medicine (EBM) extracted search terms, which all coauthors approved. It is unclear who performed the searches.	Sensitivity and precision calculations based on the relevance of the first 40 records in the search output as compared to a gold standard.	PubMed	PICO*	Median:	Median:
						17.9§, ‡‡	6.3§, ‡‡
						26.1†, ‡‡	8.8†, ‡‡
						29.6†, **	11.3†, **
						15.5†, ††	20.0†, ††
						54.7‡, ‡‡	32.1‡, ‡‡
						54.7‡, **	32.8‡, **
						15.5‡, ††	50.0‡, ††
					PIC (truncated version of PICO)*	Median:	Median:
						9.8§, ‡‡	2.5§, ‡‡
						14.6†, ‡‡	5.0†, ‡‡
						17.6†, **	5.0†, **
						48.5‡, ‡‡	21.3 ‡, ‡‡
						52.8‡, **	23.8‡, **
					PubMed link to related articles*	Median:	Median:
						39.7§, ‡‡	10.0§, ‡‡
						37.9§, ‡‡	10.0§, ‡‡
						37.5§, ‡‡	7.5§, ‡‡
Hoogendam et al., 2012 [22]	Randomized controlled crossover trial	8 specialists and 14 residents with interest in vascular medicine.	Sensitivity and precision calculations based on the relevance of all search output as compared to a gold standard.	PubMed	PICO	Average: 13.62	Average:3.44
					Unguided search	Average: 12.27	Average:4.02
Methley et al., 2014 [23]	Observational study (study design not indicated)	Search strategy developed as collaboration between some or all study authors and a specialist librarian and information specialist.	Sensitivity and precision calculations based on the relevance of all search output as assessed by the study authors.	CINAHL	PICO	77.78	1.04
				Embase		72.22	0.1
				MEDLINE		66.67	0.15
				CINAHL	PICOS	66.67	8.22
				Embase		38.88	3.7
				MEDLINE		33.33	5.32
				CINAHL	SPIDER	66.67	8.22
				Embase		16.67	5.45
				MEDLINE		27.78	35.71

* Queries were combined with a †broad therapeutic intervention filter, ‡a narrow therapeutic intervention filter, or §no filter and further limited to **English language and human studies; ††English language, human studies, and *Abridged Index Medicus* titles; or ‡‡no limitations.

Agoritsas et al. evaluated searches outlined by the authors of the study based on the PICO framework and combined into queries; although not explicitly stated, the authors likely also performed the searches [21]. The study evaluated 15 search strategies that varied in their query structure (PIC or PICO), use of PubMed’s Clinical Queries therapeutic filters (broad or narrow), and search limits, as well use of PubMed links to related articles. A total of 450 searches were performed. Relevance was assessed on the first 40 records of the search output as well as the complete search output. The study reports that the PICO model resulted in increased median sensitivity and precision of the search results.

Hoogendam et al. evaluated the effectiveness of PICO versus unguided searching among 14 residents and 8 specialists who had an interest in vascular medicine [22]. Participants received a lecture by an expert searcher explaining the basics of PubMed to ensure a basic knowledge of PubMed functionality. Participants performed unguided searching for 5 minutes on 12 therapeutic questions regarding vascular medicine. After 2 weeks, an expert searcher explained the use of PICO, and participants performed PICO searching for 5 minutes on 12 different therapeutic questions. Although not statistically significant at the *p*<0.05 level, using the PICO model resulted in a higher average sensitivity and lower average precision than did unguided searching.

Methley et al. evaluated the SPIDER conceptualizing model [23]. The authors developed a detailed search strategy in collaboration with a specialist librarian and information specialist. Identical search terms were combined using the PICO, PICOS, or SPIDER search strategy tools and compared across PubMed, Embase, and CINAHL, resulting in a total of nine searches. The authors found that PICO retrieved the largest number of hits and recommended using PICO instead of SPIDER.

### Qualitative analysis

The three included studies varied widely in their design, choice of comparators, number of databases searched, procedure for relevance assessment, and methods of calculating outcomes (Table 3).

#### Study design

One study was designed as a randomized trial including health professionals (residents and specialists) [22]; the other two were observational studies in which the authors were involved in the literature searches along with a specialist librarian and information specialist [23] or without stating who exactly performed the searches [21].

#### Relevance assessment

Two of the three included studies used Cochrane systematic reviews to formulate the clinical questions. These reviews were used as a basis for the search strategies and as a gold standard for determining the sensitivity and precision of the search results [21, 22]. One study compared PICO to PICOS and SPIDER with a focus on a specific research question; as a consequence, the search strategy was built from elements of the research question, and the relevance of search results was judged against inclusion criteria [23]. Consequently, the included studies calculated sensitivity and precision from a gold standard [21, 22] or a list of included studies [23].

#### Choice of comparator

Two of the three included studies compared the PICO model to alternative conceptualizing models. However, the two studies compared PICO to different conceptualizing models; thus, the PICO model was not compared to the same alternative conceptualizing models across studies.

One study compared the PICO model to the truncated PIC model in PubMed and reported that the PICO model resulted in increased median sensitivity and precision of the searches [21]. However, the performance of the tested search strategies was highly variable depending on the clinical question, and none of the 15 strategies showed a consistently high sensitivity in retrieving relevant articles. The study also used PubMed links to related articles as a search strategy, which resulted in higher sensitivity and precision than both the PICO and PIC models. The calculations were based on the first 40 records of the PubMed output as well as the complete search output. When the full output was screened for relevant studies, about 85% of records were detected by the PIC queries and about 69% by the PICO queries [21].

One study compared the PICO model to PICOS and SPIDER in CINAHL, Embase, and MEDLINE [23]. Although hardly conclusive due to extremely limited data, the use of PICO as a search strategy tool resulted in higher sensitivity and lower precision than the use of PICOS and SPIDER. However, as different numbers of search blocks were used for each model (i.e., PICO: 3 search blocks, PICOS: 4 search blocks, SPIDER: 6 search blocks), these results are expected.

One study compared the PICO model to unguided searching [22]. The study reported that use of the PICO model resulted in higher average sensitivity and lower average precision than did unguided searches, although this difference was not statistically significant.

#### Outcomes reported

None of the included studies investigated the time spent on the literature search.

### Risk of bias assessment

We used three risk-of-bias criteria to assess the risk of bias: (i) searcher skills, (ii) match between model and question, and (iii) quality of searches. Overall, there were several instances of unclear or high risk of bias with respect to all three criteria (Table 4). The searcher skills criterion revealed either an unclear risk of bias [21, 23] or a high risk of bias [22] in the studies. The match between model and question criterion revealed that two studies [21, 23] had a high risk of bias and one study [22] had an unclear risk of bias. Finally, we found that the quality of searches criterion revealed that two studies [21, 22] had an unclear risk of bias, and one study had a low risk of bias [23]. A complete overview of the risk of bias assessments can be found in supplementary Appendix C.

**Table 4:** Risk-of-bias summary

Study (Author, year)	Searcher skills	Fit between model and topic	Quality of searches
Agoritsas et al., 2012 [21]	Unclear	High	Unclear
Hoogendam et al., 2012 [22]	High	Unclear	Unclear
Methley et al., 2014 [23]	Unclear	High	Low

## DISCUSSION

This study is the first systematic review aiming to determine whether the use of the PICO model as search strategy tool affects the quality of the literature search, which had the potential to provide valuable evidence of the effect of using PICO to formulate search queries. This review is strengthened by the use of rigorous methods based on prespecified criteria in a protocol following both the AMSTAR measurement tool [14] and PRISMA 2009 checklist [15], a comprehensive literature search and duplicate screening process, data extraction, and risk-of-bias assessment. However, we identified only three studies that were eligible for inclusion in the review [21–23], and given the marked differences among studies, it was only possible to perform qualitative analysis.

Despite the rigorous methodology that we used, there are limitations for this review. No validated assessment tool exists for these types of studies, which led us to develop our own set of risk-of-bias criteria. As opposed to validated criteria such as Cochrane’s risk-of-bias tool for assessing randomized trials [24], our tool was not validated, which would have been preferable. Despite the limitations of our risk-of-bias tool, we regarded all three included studies [21–23] as having a high or unclear risk of bias. Consequently, it is extremely difficult to draw any conclusions from their findings.

As no similar reviews exist, we turn to the individual studies to enlighten our discussion on whether the use of the PICO model as search strategy tool affects the quality of the literature search. Two issues are prominent: the importance of the number of search blocks and the practice of avoiding outcome-related terms in the search strategy.

First, the number of search blocks in a literature search is important for the search output. That is, the more search blocks that are included, the more restricted the search output will be. One of the included studies did not compensate for the number of search blocks in each strategy, and thus, as expected, the search strategy tool with the lowest number of blocks retrieved a greater number of hits [23]. Existing guidelines recommend using only the truncated PIC version of the PICO model for performing literature searches for systematic reviews [13]. The rationale is that some or all outcome measures might not be mentioned in abstracts, and including a search block defining the outcomes leads to a lower sensitivity of the literature search.

One study that was included in this review investigated the median sensitivity and precision of the PICO model compared to the PIC model [21]. Surprisingly, the study reported that the PICO model performed better than the truncated PIC model with regard to sensitivity and precision. However, these results were based only on the first forty records of the search output, which might explain this surprising finding, because an inverse relationship usually exists between sensitivity and precision [18]. Also, depending on how the search results were sorted, different results could be obtained. When considering the full search output, the PIC model did show a higher sensitivity and lower precision, although both measures varied greatly across different searches [21]. This finding of higher sensitivity and lower precision when using the PIC model (three search blocks) compared with the PICO model (four search blocks) [21] is in accordance with another included study that found that the PICO model (using three search blocks: P, I, and O) resulted in higher sensitivity and lower precision than the PICOS model (four search blocks) or SPIDER model (five search blocks) [23]. Taken together, these results suggest that the number of search blocks impacts the quality of the search output as quantified by sensitivity and precision.

Second, the claim that searching for outcome-related terms when using the PICO model as a search strategy tool lowers the sensitivity of the search [13] is not substantiated. Based on the limited data from this review, however, we are not able to make any firm conclusions. The study addressing this issue [21] focused on identifying search components and tools that could help clinicians build more effective strategies to answer questions at the point of care and did not include sophisticated strategies used for performing systematic reviews; thus, its results are of limited generalizability. Future studies investigating the effect of searching for outcome-related terms are needed to support this recommendation [10].

The PICO model was developed to help structure a well-built clinical question and enable a literature search [6]. Since its introduction, it has played an important role as a conceptualizing model in EBM [10]. However, evidence of the effect of using the PICO model as a search strategy tool is still lacking, and the studies that were included in this review do not allow us to build upon this important body of evidence. To practice EBM with evidence-based methods, and thus ensure rigorous methodology, the results of this review indicate that more work is needed to assess the applicability of specific conceptualizing models. Furthermore, we propose that it is important for future research on this topic to address three potential risks of bias: (i) searcher skills, (ii) match between model and question, and (iii) quality of searches.

Overall, there have been few studies assessing the effect of using the PICO model versus other available models or unguided searching on the quality of literature search results. Specifically, despite a rigorous search and selection process, we found only three such studies. Due to heterogeneity among these studies, quantitative analysis was not possible, and no solid conclusions about the effect of using the PICO model on the quality of the literature search could be drawn. Before implications for current practice can be made, there is a need for well-designed studies to evaluate the role of the tool used to devise a search strategy.

## SUPPLEMENTAL FILES

Appendix A Literature searches

Appendix B Excluded studies

Appendix C Data extraction form and risk of bias assessment
