# Supplementary material for: The impact of patient, intervention, comparison, outcome (PICO) as a search strategy tool on literature search quality: a systematic review
Source: J Med Libr Assoc. 2018 Oct 1;106(4):420–31. doi: 10.5195/jmla.2018.345 (PMC6148624; doi:10.5195/jmla.2018.345)
Supplement: Appendix A [file jmla-106-420-s001.pdf]

JMLA Journal of the Medical Library Association

SC

Supplemental content to
J Med Libr Assoc. Oct;106(4):dx.doi.org/10.5195/jmla.2018.345
www.jmla.mlanet.org
© Eriksen, Frandsen 2018

The impact of patient, intervention, comparison, outcome (PICO) as a search strategy tool on literature search quality: a systematic review

Mette Brandt Eriksen, PhD; Tove Faber Frandsen, PhD

APPENDIX A

Literature searches

PubMed

Search: January 9, 2017

	Search	Result
#1	"databases, bibliographic"[MeSH Terms] OR "Computer Literacy" [MeSH] OR "Data mining" [MeSH] OR "Evidence Based Dentistry" [MeSH] OR "Evidence-Based Emergency Medicine" [MeSH] OR "Evidence-based Medicine" [MeSH] OR "Evidence-based Nursing" [MeSH] OR "Evidence Based Practice" [MeSH] OR "Health literacy" [MeSH] OR "Information literacy" [MeSH] OR "literature based discovery" [MeSH] OR "information seeking behavior" [MeSH] "information storage and retrieval" [MeSH] OR "data mining" [MeSH] OR Bibliographic database search [All Fields] OR Bibliographic database searches [All Fields] OR Bibliographic database searching [All Fields] OR Bibliographic databases search [All Fields] OR Bibliographic databases searches [All Fields] OR Bibliographic databases searching [All Fields] OR Computer literacies [All Fields] OR Computer Literacy [All Fields] OR Computerized Literature Searching [All Fields] OR Data file [All Fields] OR Data files [All Fields] OR Data linkage [All Fields] OR Data mining [All Fields] OR Data retrieval [All Fields] OR Data retrieving [All Fields] OR Data source [All Fields] OR Data sources [All Fields] OR Data storage [All Fields] OR Datamining [All Fields] OR Evidence Based Dental Practice [All Fields] OR Evidence Based Dentistries [All Fields] OR Evidence Based Dentistry [All Fields] OR Evidence Based Emergency Medicine [All Fields] OR Evidence based emergency medicines [All Fields] OR Evidence based health care [All Fields] OR Evidence Based Healthcare [All Fields] OR Evidence based healthcares [All Fields] OR Evidence Based Medical Practice [All Fields] OR Evidence Based Medicine [All Fields] OR Evidence Based Nursing [All Fields] OR Evidence Based Practice [All Fields] OR Evidence based professional practice [All Fields] OR Health literacies [All Fields] OR Health literacy [All Fields] OR Information extraction [All Fields] OR Information extractions [All Fields] OR Information literacies [All Fields] OR Information literacy [All Fields] OR Information processing [All Fields] OR Information retrieval [All Fields] OR Information retrieving [All Fields] OR Information seeking behavior [All Fields] OR Information storage [All Fields] OR literature based discovery [All Fields] OR literature retrieval [All Fields] OR Literature retrieving [All Fields] OR Literature search [All Fields] OR Literature searches [All Fields] OR Literature Searching [All Fields] OR Machine readable data file [All Fields] OR Machine readable data files [All Fields] OR Online database search [All Fields] OR Online database searches [All Fields] OR Online database searching [All Fields] OR Online databases search [All Fields] OR Online databases searches [All Fields] OR Online databases searching [All Fields] OR Research Based Medical Practice [All Fields] OR Research Based Nursing Practice [All Fields] OR Research Based Occupational Therapy Practice [All Fields] OR Research Based Physical Therapy Practice [All Fields] OR Research Based Professional Practice [All Fields] OR Review Literature as Topic [All Fields] OR Search strategies [All Fields] OR Search strategy [All Fields] OR State of the art review [All Fields] OR State of the art reviews [All Fields] OR Systematic review topic [All Fields] OR Text mining [All Fields] OR Theory Based Nursing Practice [All Fields]	2,846,396
#2	Pico [All Fields] OR patient intervention comparison outcome [All Fields] OR patient intervention comparator outcome [All Fields] OR (population intervention comparison outcome [All Fields] OR population intervention comparison outcomes [All Fields]) OR problem intervention comparison outcome [All Fields]	2,747
#3	#1 AND #2	601

Embase (Embase Classic+Embase 1947 to 6 January 20176)
Search: January 9, 2017

	Search	Result
#1	Information retrieval/	31,231
#2	Information seeking/	2,051
#3	Information storage/	1,636
#4	Data mining/	9,496
#5	exp evidence based practice/	1,058,808
#6	Health literacy/	5,984
#7	Information literacy/	348
#8	Information retrieval/	31,231
#9	((Bibliographic adj3 database* adj3 search*) or (Computer* adj3 literac*) or (Computerized adj3 Literature adj3 Search*) or (Data adj3 fil*) or (Data adj3 linkage) or (Data adj3 mining) or Datamining or (Data adj3 retriev*) or (Data adj3 sourc*) or (Data adj3 storage*) or (Evidence adj3 Based adj3 Dent*) or (Evidence adj3 Based adj3 Emerg* Med*) or (Evidence adj3 Based adj3 Healthcar*) or (Evidence adj3 based adj3 health adj3 care*) or (Evidence adj3 Based adj3 Med*) or (Evidence adj3 Based adj3 Nurs*) or (Evidence adj3 Based adj3 Pract*) or (Health adj3 literac*) or (Information adj3 extraction*) or (Information adj3 literac*) or (Information adj3 process*) or (information adj3 retriev*) or (Information adj3 seek*) or (Information adj3 stor*) or (literature adj3 based adj3 discovery) or (literature adj3 retriev*) or (Literature adj3 search*) or (Machine adj3 readable adj3 data file*) or (Online adj3 database* adj3 search*) or (Research adj3 based adj3 practice) or (Review adj3 Literature adj3 Topic) or (Search adj3 strateg*) or (State of the art adj3 review*) or (Systematic adj3 review adj3 topic*) or (Text adj3 mining) or (Theory adj3 Based adj3 Nurs* adj3 Practice)).mp. [mp=title, abstract, heading word, drug trade name, original title, device manufacturer, drug manufacturer, device trade name, keyword, floating subheading]	635,895
#10	#1–9/OR	1,452,064
#11	(Pico or (patient adj3 intervention adj3 comparison adj3 outcome*) or (Patient adj3 Intervention adj3 Comparator adj3 Outcome*) or (population adj3 intervention adj3 comparison adj3 outcome*) or (problem adj3 intervention adj3 comparison adj3 outcome*)).mp. [mp=title, abstract, heading word, drug trade name, original title, device manufacturer, drug manufacturer, device trade name, keyword, floating subheading]	2,037
#12	#10 AND #11 (Saved as "PICO review complete search" searched January 2, 2017)	611

JMLA Journal of the Medical Library Association

Supplemental content to
J Med Libr Assoc. Oct;106(4):dx.doi.org/10.5195/jmla.2018.345
www.jmla.mlanet.org
© Eriksen, Frandsen 2018

Cochrane Library (Wiley Online Library)

Search: January 9, 2017

	Search	Result
#1	MeSH descriptor: [Information Storage and Retrieval] explode all trees	1,578
#2	MeSH descriptor: [Information Literacy] explode all trees	212
#3	MeSH descriptor: [Automatic Data Processing] explode all trees	105
#4	MeSH descriptor: [Review Literature as Topic] explode all trees	162
#5	MeSH descriptor: [Computer Literacy] explode all trees	44
#6	MeSH descriptor: [Data Mining] explode all trees	17
#7	MeSH descriptor: [Evidence-Based Dentistry] explode all trees	33
#8	MeSH descriptor: [Evidence-Based Emergency Medicine] explode all trees	5
#9	MeSH descriptor: [Evidence-Based Practice] explode all trees	2,429
#10	MeSH descriptor: [Evidence-Based Medicine] explode all trees	2,101
#11	MeSH descriptor: [Evidence-Based Nursing] explode all trees	48
#12	MeSH descriptor: [Health Literacy] explode all trees	209
#13	MeSH descriptor: [Information Seeking Behavior] explode all trees	36
#14	MeSH descriptor: [Literature Based Discovery] explode all trees	0
#15	(Bibliographic near/3 database* near/3 search*) or (Computer* near/3 literac*) or (Computerized near/3 Literature near/3 Search*) or (Data near/3 fil*) or (Data near/3 linkage) or (Data near/3 mining) or Datamining or (Data near/3 retriev*) or (Data near/3 sourc*) or (Data near/3 storage*) or (Evidence near/3 Based near/3 Dent*) or (Evidence near/3 Based near/3 Emerg* Med*) or (Evidence near/3 Based near/3 Healthcar*) or (Evidence near/3 based near/3 health near/3 care*) or (Evidence near/3 Based near/3 Med*) or (Evidence near/3 Based near/3 Nurs*) or (Evidence near/3 Based near/3 Pract*) or (Health near/3 literac*) or (Information near/3 extraction*) or (Information near/3 literac*) or (Information near/3 process*) or (information near/3 retriev*) or (Information near/3 seek*) or (Information near/3 stor*) or (literature near/3 based near/3 discovery) or (literature near/3 retriev*) or (Literature near/3 search*) or (Machine near/3 readable near/3 data file*) or (Online near/3 database* near/3 search*) or (Research near/3 based near/3 practice) or (Review near/3 Literature near/3 Topic) or (Search near/3 strateg*) or (State of the art near/3 review*) or (Systematic near/3 review near/3 topic*) or (Text near/3 mining) or (Theory near/3 Based near/3 Nurs* near/3 Practice):ti,ab,kw (Word variations have been searched)	15,428
#16	#1–#15/OR	16,850
#17	Pico or (patient near/3 intervention near/3 comparison near/3 outcome*) or (Patient near/3 Intervention near/3 Comparator near/3 Outcome*) or (population near/3 intervention near/3 comparison near/3 outcome*) or (problem near/3 intervention near/3 comparison near/3 outcome*):ti,ab,kw (Word variations have been searched)	68
#18	#16 AND #17	24

Supplemental content to
J Med Libr Assoc. Oct;106(4):dx.doi.org/10.5195/jmla.2018.345
www.jmla.mlanet.org
© Eriksen, Frandsen 2018

PsycINFO (PsycINFO 1806 to January, Week 1, 2017)
Search: January 9, 2017

	Search	Result
#1	exp Data Mining/	1,733
#2	exp Evidence Based Practice/	14,239
#3	exp Health Literacy/	1,967
#4	exp Information Literacy/	229
#5	exp computer literacy/	534
#6	((Bibliographic adj3 database* adj3 search*) or (Computer* adj3 literac*) or (Computerized adj3 Literature adj3 Search*) or (Data adj3 fil*) or (Data adj3 linkage) or (Data adj3 mining) or Datamining or (Data adj3 retriev*) or (Data adj3 sourc*) or (Data adj3 storage*) or (Evidence adj3 Based adj3 Dent*) or (Evidence adj3 Based adj3 Emerg* Med*) or (Evidence adj3 Based adj3 Healthcar*) or (Evidence adj3 based adj3 health adj3 care*) or (Evidence adj3 Based adj3 Med*) or (Evidence adj3 Based adj3 Nurs*) or (Evidence adj3 Based adj3 Pract*) or (Health adj3 literac*) or (Information adj3 extraction*) or (Information adj3 literac*) or (Information adj3 process*) or (information adj3 retriev*) or (Information adj3 seek*) or (Information adj3 stor*) or (literature adj3 based adj3 discovery) or (literature adj3 retriev*) or (Literature adj3 search*) or (Machine adj3 readable adj3 data file*) or (Online adj3 database* adj3 search*) or (Research adj3 based adj3 practice) or (Review adj3 Literature adj3 Topic) or (Search adj3 strateg*) or (State of the art adj3 review*) or (Systematic adj3 review adj3 topic*) or (Text adj3 mining) or (Theory adj3 Based adj3 Nurs* adj3 Practice)).mp. [mp=title, abstract, heading word, table of contents, key concepts, original title, tests & measures]	116,470
#7	#1–#6/ OR	116,470
#8	Pico or (patient adj3 intervention adj3 comparison adj3 outcome*) or (Patient adj3 Intervention adj3 Comparator adj3 Outcome*) or (population adj3 intervention adj3 comparison adj3 outcome*) or (problem adj3 intervention adj3 comparison adj3 outcome*)	87
#9	#7 AND #8	32

JMLA Journal of the Medical Library Association

SC

Supplemental content to
J Med Libr Assoc. Oct;106(4):dx.doi.org/10.5195/jmla.2018.345
www.jmla.mlanet.org
© Eriksen, Frandsen 2018

CINAHL (via EBSCO Host)

Search: January 9, 2017

	Search	Result
#1	(MH "Computer Literacy")	864
#2	(MH "Data Mining")	1,106
#3	(MH "Evidence-Based Dental Practice")	240
#4	(MH "Nursing Practice, Evidence-Based+")	10,410
#5	(MH "Professional Practice, Evidence-Based+")	45,961
#6	(MH "Information Literacy+")	3,217
#7	(MH "Information Retrieval+")	6,575
#8	(MH "Information Seeking Behavior")	2,369
#9	(MH "Information Storage+")	5,113
#10	(MH "Literature Searching+")	6,827
#11	(MH "Professional Practice, Research-Based+")	4,247
#12	TX (Bibliographic N4 database* N4 search*) or (Computer* N4 literac*) or (Computerized N4 Literature N4 Search*) or (Data N4 fil*) or (Data N4 linkage) or (Data N4 mining) or Datamining or (Data N4 retriev*) or (Data N4 sourc*) or (Data N4 storage*) or (Evidence N4 Based N4 Dent*) or (Evidence N4 Based N4 Emerg* Med*) or (Evidence N4 Based N4 Healthcar*) or (Evidence N4 based N4 health N4 care*) or (Evidence N4 Based N4 Med*) or (Evidence N4 Based N4 Nurs*) or (Evidence N4 Based N4 Pract*) or (Health N4 literac*) or (Information N4 extraction*) or (Information N4 literac*) or (Information N4 process*) or (information N4 retriev*) or (Information N4 seek*) or (Information N4 stor*) or (literature N4 based N4 discovery) or (literature N4 retriev*) or (Literature N4 search*) or (Machine N4 readable N4 data file*) or (Online N4 database* N4 search*) or (Research N4 based N4 practice) or (Review N4 Literature N4 Topic) or (Search N4 strateg*) or (State of the art N4 review*) or (Systematic N4 review N4 topic*) or (Text N4 mining) or (Theory N4 Based N4 Nurs* N4 Practice)	156,452
#13	#1 - #12/OR	160,393
#14	TX (pico or (patient N4 intervention N4 comparison N4 outcome*) or (Patient N4 Intervention N4 Comparator N4 Outcome*) or (population N4 intervention N4 comparison N4 outcome*) or (problem N4 intervention N4 comparison N4 outcome*))	323
#15	#13 AND #14	146

JMLA Journal of the Medical Library Association

Supplemental content to
J Med Libr Assoc. Oct;106(4):dx.doi.org/10.5195/jmla.2018.345
www.jmla.mlanet.org
© Eriksen, Frandsen 2018

Library and Information Science Abstracts (LISA) (via ProQuest)
Search: January 9, 2017

	Search	Result
#1	SU.EXACT("Data mining")	1,422
#2	SU.EXACT("Evidence-based medicine")	471
#3	SU.EXACT("Evidence-based nursing")	8
#4	SU.EXACT("Information literacy")	4,782
#5	SU.EXACT("Information processing")	37
#6	SU.EXACT("Information retrieval")	1,403
#7	SU.EXACT("Information seeking behavior")	238
#8	SU.EXACT("Information storage")	27
#9	Anywhere ((Bibliographic database* search*) OR (Computer* literac*) OR (Computerized Literature Search*) OR (Data fil*) OR (Data linkage) OR (Data mining) OR determining OR (Data retriev*) OR (Data sourc*) OR (Data storage*) OR (Evidence Based Dent*) OR (Evidence Based Emerg* Med*) OR (Evidence Based Healthcar*) OR (Evidence based health care*) OR (Evidence Based Med*) OR (Evidence Based Nurs*) OR (Evidence Based Pract*) OR (Health literac*) OR (Information extraction*) OR (Information literac*) OR (Information process*) OR (information retriev*) OR (Information seek*) OR (Information stor*) OR (literature based discovery) OR (literature retriev*) OR (Literature search*) OR (Machine readable data file*) OR (Online database* search*) OR (Research based practice) OR (Review Literature Topic) OR (Search strateg*) OR (State of the art review*) OR (Systematic review topic*) OR (Text mining) OR (Theory Based Nurs* Practice))	137,088
#10	#1 - #9/OR	137,149
#11	Anywhere (pico or (patient intervention comparison outcome*) or (Patient Intervention Comparator Outcome*) or (population intervention comparison outcome*) or (problem intervention comparison outcome*))	42
#12	#11 and #12	26

SC
Supplemental content to
J Med Libr Assoc. Oct;106(4):dx.doi.org/10.5195/jmla.2018.345
www.jmla.mlanet.org
© Eriksen, Frandsen 2018

National Library of Medicine (NLM) catalog

Search: January 9, 2017

	Search	Result
#1	Bibliographic database search OR Bibliographic database searches OR Bibliographic database searching OR Bibliographic databases search OR Bibliographic databases searches OR Bibliographic databases searching OR Computer literacies OR Computer Literacy OR Computerized Literature Searching OR Data file OR Data files OR Data linkage OR Data mining OR Data retrieval OR Data retrieving OR Data source OR Data sources OR Data storage OR Datamining OR Evidence Based Dental Practice OR Evidence Based Dentistries OR Evidence Based Dentistry OR Evidence Based Emergency Medicine OR Evidence based emergency medicines OR Evidence based health care OR Evidence Based Healthcare OR Evidence based healthcares OR Evidence Based Medical Practice OR Evidence Based Medicine OR Evidence Based Nursing OR Evidence Based Practice OR Evidence based professional practice OR Health literacies OR Health literacy OR Information extraction OR Information extractions OR Information literacies OR Information literacy OR Information processing OR Information retrieval OR Information retrieving OR Information seeking behavior OR Information storage OR literature based discovery OR literature retrieval OR Literature retrieving OR Literature search OR Literature searches OR Literature Searching OR Machine readable data file OR Machine readable data files OR Online database search OR Online database searches OR Online database searching OR Online databases search OR Online databases searches OR Online databases searching OR Research Based Medical Practice OR Research Based Nursing Practice OR Research Based Occupational Therapy Practice OR Research Based Physical Therapy Practice OR Research Based Professional Practice OR Review Literature as Topic OR Search strategies OR Search strategy OR State of the art review OR State of the art reviews OR Systematic review topic OR Text mining OR Theory Based Nursing Practice	31,244
#2	pico OR patient intervention comparison outcome* OR Patient Intervention Comparator Outcome* OR population intervention comparison outcome* OR problem intervention comparison outcome*	46
#3	#1 AND #2	13

Supplemental content to
J Med Libr Assoc. Oct;106(4):dx.doi.org/10.5195/jmla.2018.345
www.jmla.mlanet.org
© Eriksen, Frandsen 2018

Scopus (Elsevier)

Search: January 9, 2017

	Search	Result
#1	TITLE-ABS-KEY (bibliographic W/3 database* W/3) OR (Computer* W/3 literac*) OR (Computerized W/3 Literature W/3 Search*) OR (Data W/3 fil*)	59,744
#2	TITLE-ABS-KEY (Data W/3 linkage) or (Data W/3 mining) or Datamining or (Data W/3 retriev*) or (Data W/3 sourc*) or (Data W/3 storage*)	343,795
#3	TITLE-ABS-KEY (Evidence W/3 Based W/3 Dent*) or (Evidence W/3 Based W/3 Emerg* Med*) or (Evidence W/3 Based W/3 Healthcar*) or (Evidence W/3 based W/3 health W/3 care*) or (Evidence W/3 Based W/3 Med*)	124,308
#4	TITLE-ABS-KEY (Evidence W/3 Based W/3 Nurs*) or (Evidence W/3 Based W/3 Pract*) or (Health W/3 literac*) or (Information W/3 extraction*) or (Information W/3 literac*) or (Information W/3 process*) or (information W/3 retriev*)	531,542
#5	TITLE-ABS-KEY (Information W/3 seek*) or (Information W/3 stor*) or (literature W/3 based W/3 discovery) or (literature W/3 retriev*) or (Literature W/3 search*) or (Machine W/3 readable W/3 data file*)	118,361
#6	TITLE-ABS-KEY (Online W/3 database* W/3 search*) or (Research W/3 based W/3 practice) or (Review W/3 Literature W/3 Topic) or (Search W/3 strateg*) or (State of the art W/3 review*)	46,984
#7	TITLE-ABS-KEY (Systematic W/3 review W/3 topic*) or (Text W/3 mining) or (Theory W/3 Based W/3 Nurs* W/3 Practice)	21,355
#8	#1–#7/OR	1,123,581
#9	TITLE-ABS-KEY (pico OR (patient W/3 intervention W/3 comparison W/3 outcome*) OR (patient W/3 intervention W/3 comparator W/3 outcome*) OR (population W/3 intervention W/3 comparison W/3 outcome*) OR (problem W/3 intervention W/3 comparison W/3 outcome*))	8,694
#10	#8 AND #9	454

JMLA Journal of the Medical Library Association

SC

Supplemental content to
J Med Libr Assoc. Oct;106(4):dx.doi.org/10.5195/jmla.2018.345
www.jmla.mlanet.org
© Eriksen, Frandsen 2018

Web of Science (Web of Science Core Collection, Thompson Reuters)
Search: January 9, 2017

	Search	Result
#1	TS=(Bibliographic NEAR/3 database* NEAR/3 search*) or (Computer* NEAR/3 literac*) or (Computerized NEAR/3 Literature NEAR/3 Search*) or (Data NEAR/3 fil*) or (Data NEAR/3 linkage) or (Data NEAR/3 mining) or Datamining or (Data NEAR/3 retriev*) or (Data NEAR/3 sourc*) or (Data NEAR/3 storage*) or (Evidence NEAR/3 Based NEAR/3 Dent*) or (Evidence NEAR/3 Based NEAR/3 Emerg* Med*) or (Evidence NEAR/3 Based NEAR/3 Healthcar*) or (Evidence NEAR/3 based NEAR/3 health NEAR/3 care*) or (Evidence NEAR/3 Based NEAR/3 Med*) or (Evidence NEAR/3 Based NEAR/3 Nurs*) or (Evidence NEAR/3 Based NEAR/3 Pract*) or (Health NEAR/3 literac*) or (Information NEAR/3 extraction*) or (Information NEAR/3 literac*) or (Information NEAR/3 process*) or (information NEAR/3 retriev*) or (Information NEAR/3 seek*) or (Information NEAR/3 stor*) or (literature NEAR/3 based NEAR/3 discovery) or (literature NEAR/3 retriev*) or (Literature NEAR/3 search*) or (Machine NEAR/3 readable NEAR/3 data file*) or (Online NEAR/3 database* NEAR/3 search*) or (Research NEAR/3 based NEAR/3 practice) or (Review NEAR/3 Literature NEAR/3 Topic) or (Search NEAR/3 strateg*) or (State of the art NEAR/3 review*) or (Systematic NEAR/3 review NEAR/3 topic*) or (Text NEAR/3 mining) or (Theory NEAR/3 Based NEAR/3 Nurs* NEAR/3 Practice)<br>*Indexes=SCI-EXPANDED, SSCI, A&HCI, CPCI-S, CPCI-SSH, ESCI Timespan=All years*	455,401
#2	TS=(pico or (patient NEAR/3 intervention NEAR/3 comparison NEAR/3 outcome*) or (Patient NEAR/3 Intervention NEAR/3 Comparator NEAR/3 Outcome*) or (population NEAR/3 intervention NEAR/3 comparison NEAR/3 outcome*) or (problem NEAR/3 intervention NEAR/3 comparison NEAR/3 outcome*))	5,542
#3	#1 AND #2	256
