# Supplementary material for: The impact of patient, intervention, comparison, outcome (PICO) as a search strategy tool on literature search quality: a systematic review
Source: J Med Libr Assoc. 2018 Oct 1;106(4):420–31. doi: 10.5195/jmla.2018.345 (PMC6148624; doi:10.5195/jmla.2018.345)
Supplement: Appendix B [file jmla-106-420-s002.pdf]

JMLA Journal of the Medical Library Association

SC
Supplemental content to
J Med Libr Assoc. Oct;106(4):dx.doi.org/10.5195/jmla.2018.345
www.jmla.mlanet.org
© Eriksen, Frandsen 2018

The impact of patient, intervention, comparison, outcome (PICO) as a search strategy tool on literature search quality: a systematic review

Mette Brandt Eriksen, PhD; Tove Faber Frandsen, PhD

APPENDIX B

Excluded studies

The following studies were excluded from the review due to wrong study design, wrong intervention, or wrong outcomes.

Wrong study design

- Boudin F, Nie JY, Bartlett JC, Grad R, Pluye P, Dawes M. Combining classifiers for robust PICO element detection. BMC Med Inform Decis Mak. 2010 May 15;10:29.
- Bramer WM. Patient, intervention, control, and outcome (PICO): an overrated tool [expert searching]. MLA News. 2015 Feb;55(2):6.
- Chillingworth M. Pico reveals latent talent. Inf World Rev. 2006;(224):33.
- da Costa Santos CM1, de Mattos Pimenta CA, Nobre MR. The PICO strategy for the research question construction and evidence search. Rev Lat Am Enfermagem. 2007 May–Jun;15(3):508–11.
- Davies KS. Formulating the evidence based practice question: a review of the frameworks. Evidence Based Libr Inf Pract. 2011;6(2):75–80.
- Diaz M, Chacón JDO, Ronda VM, José F. Design of clinical questions in evidence-based practice. formulation models. Enfermeria Global. 2016;15(3):431–8.
- Elkins MY. Using PICO and the brief report to answer clinical questions. Nursing. 2010 Apr;40(4):59–60.
- Haroon M, Phillips R. "There is nothing like looking, if you want to find something" - asking questions and searching for answers - the evidence based approach. Arch Dis Child Educ Pract Ed. 2010 Apr;95(2):34–9.
- Hastings C, Fisher CA. Searching for proof: creating and using an actionable PICO question. Nurs Manage. 2014 Aug;45(8):9–12.
- Henley M. Using patient characteristics, interventions, comparison group, and expected (or desired) outcomes (PICO) for clinical questions: simple search strategies for finding best evidence [expert searching]. MLA News. 2013 Sep;53(8):10.
- Medina McKeon JM, McKeon PO. PICO: a hot topic in evidence-based practice. Int J Athl Ther Train. 2015 Jan;20(1):1–3.
- Miller SA, Forrest JL. Enhancing your practice through evidence-based decision making: PICO, learning how to ask good questions. J Evidence-Based Dental Pract. 2001 Oct;1(2):136–41.
- Stone PW. Popping the (PICO) question in research and evidence-based practice [ask an expert]. Appl Nurs Res. 2002 Aug;15(3):197–8.
- Sun H, Shi JP. Expansion of PICO model in evidence-based medicine and its application in qualitative research. Chin J Evidence-Based Med. 2014 Jan;14(5):505–8.
- Van Den Bruel A, Boland B, Vermeire E, Buntinx F, Aertgeerts B. [From PICO to search terms on the Internet: how to find relevant information? new coxibs: do they have a better gastrointestinal safety?] Rev Med de Liege. 2005 Jan;60(1):52–60.
- Yensen J. PICO search strategies. Online J Nurs Inform. 2013 Fall;17(3):1–5.

JMLA Journal of the Medical Library Association

SC
Supplemental content to
J Med Libr Assoc. Oct;106(4):dx.doi.org/10.5195/jmla.2018.345
www.jmla.mlanet.org
© Eriksen, Frandsen 2018

Wrong interventions

- Schardt C, Adams MB, Owens T, Keitz S, Fontelo P. Utilization of the PICO framework to improve searching PubMed for clinical questions. BMC Med Inform Decis Mak. 2007 Jun 15;7:16.

Wrong outcomes

- Cooke A, Smith D, Booth A. Beyond PICO: the SPIDER tool for qualitative evidence synthesis. Qual Health Res. 2012 Oct;22(10):1435–43.
- Ho GJ, Liew SM, Ng CJ, Shunmugam RH, Glasziou P. Development of a search strategy for an evidence based retrieval service. PLOS ONE. 2016;11(12):e0167170.
