# Supplementary material for: The impact of patient, intervention, comparison, outcome (PICO) as a search strategy tool on literature search quality: a systematic review
Source: J Med Libr Assoc. 2018 Oct 1;106(4):420–31. doi: 10.5195/jmla.2018.345 (PMC6148624; doi:10.5195/jmla.2018.345)
Supplement: Appendix C [file jmla-106-420-s003.pdf]

JMLA Journal of the Medical Library Association

SC

Supplemental content to
J Med Libr Assoc. Oct;106(4):dx.doi.org/10.5195/jmla.2018.345
www.jmla.mlanet.org
© Eriksen, Frandsen 2018

The impact of patient, intervention, comparison, outcome (PICO) as a search strategy tool on literature search quality: a systematic review

Mette Brandt Eriksen, PhD; Tove Faber Frandsen, PhD

APPENDIX C

Data extraction form and risk of bias assessment

Data extraction form

Cochrane's data collection forms for intervention reviews, randomized controlled trials (RCTs), and non-RCTs [1] were used as guides for developing this data extraction form.

General information

Date form completed *(dd/mm/yyyy)*	05/11/2017
Name/ID of person extracting data	Consensus: Frandsen & Eriksen
Report title<br>*(title of paper/abstract/report that data are extracted from)*	Sensitivity and Predictive Value of 15 PubMed Search Strategies to Answer Clinical Questions Rated Against Full Systematic Reviews
Report ID<br>*(if there are multiple reports of this study)*	Agoritsas et al.
Reference details	Agoritsas T, Merglen A, Courvoisier DS, Combescure C, Garin N, Perrier A, Perneger TV. J Med Internet Res. 2012 Jun 12;14(3):e85.
Report author contact details	Thomas Agoritsas: Thomas.agoritsas@gmail.com
Publication type<br>*(e.g. full report, abstract, letter)*	Full report, journal article
Conflict of interest	None declared. (pg. 12)

JMLA Journal of the Medical Library Association

Supplemental content to
J Med Libr Assoc. Oct;106(4):dx.doi.org/10.5195/jmla.2018.345
www.jmla.mlanet.org
© Eriksen, Frandsen 2018

Methods

Study characteristics	Review inclusion criteria<br>*All study types were included in the review*	Yes/No/Unclear	Location in text<br>*(pg. & ¶/fig/table)*
Type of study	Randomised trial	No	
	Non-randomised trial	No	
	Controlled before-after study<br>• Contemporaneous data collection<br>• At least 2 intervention and 2 control clusters	No	
	Interrupted time series OR<br>Repeated measures study<br>• At least 3 timepoints before and 3 after the intervention<br>• Clearly defined intervention point	No	
	Other design (specify):	Observational study	Methods section does not indicate any randomization, time series or other design

JMLA Journal of the Medical Library Association

Supplemental content to
J Med Libr Assoc. Oct;106(4):dx.doi.org/10.5195/jmla.2018.345
www.jmla.mlanet.org
© Eriksen, Frandsen 2018

	**Descriptions as stated in report/paper**	**Location in text** ***(pg. & ¶/fig/table)***
Aim of study	Our aim was to identify search components and tools that would most likely help clinicians answer questions on therapeutic interventions at the point of care.	Introduction (end)
Participants<br>*No. and description of participants*	Two authors (Agoritsas and Merglen) trained in epidemiology and evidence-based medicine extracted search terms, which all coauthors finally approved.<br>Thus, the authors planned the searches. It is unclear who performed the searches.	pg. 3, Methods section states that two of the authors planned the searches. Methods section as well as "Study Limitations and Strengths" indicate that the authors performed the searches. However, it is not clearly stated.<br>pg. 3, "Extraction of Search Terms and Formulation of PICO Query"
Models included in the study<br>*Verbatim extraction*	The complete PICO query and they state that they use the truncated PIC query. However, from multimedia Table 3, it seems that PIO is rather used. The PubMed clinical queries filter is also used on all models. Searches are however also performed without filtering.	pg. 4, "Design of Search Strategies," Table 2, Figure 4
Number and wording of clinical questions included in the study	15 strategies on 30 clinical questions resulting in 450 searches.<br>The clinical questions are based on 30 Cochrane reviews from 15 different Cochrane Groups (multimedia Appendix 1). It is not clear whether the clinical questions are exactly the title of the Cochrane reviews or based on the aim of the reviews.	pg. 3, "Sample of Systematic Reviews for the Identification of Relevant Articles." "Analysis of Search Performance": first sentence

JMLA Journal of the Medical Library Association

SC

Supplemental content to
J Med Libr Assoc. Oct;106(4):dx.doi.org/10.5195/jmla.2018.345
www.jmla.mlanet.org
© Eriksen, Frandsen 2018

Outcomes

	**Description as stated in report/paper**	**Location in text** *(pg. & ¶/fig/table)*
Outcome name(s)	Sensitivity (or recall): relevant studies (included in the systematic review) retrieved by the search divided by relevant studies included in the systematic review.<br>PPV (or precision): relevant studies (included in the systematic reviews) retrieved by the search divided by the number of items generated (or screened) in the output.	pg. 5, Figure 2.<br>"Analysis of Search Performance": first paragraph
Outcome definition of relevance	Relevance is determined by inclusion in a specific Cochrane review.	"Analysis of Search Performance"

JMLA Journal of the Medical Library Association

Supplemental content to
J Med Libr Assoc. Oct;106(4):dx.doi.org/10.5195/jmla.2018.345
www.jmla.mlanet.org
© Eriksen, Frandsen 2018

<table>
<tr><th></th><th>Description as stated in report/paper</th><th>Location in text
(pg. & ¶/fig/table)</th></tr>
<tr><td>Data on precision
(indicate how calculated)</td><td>The proportion of relevant papers using a Cochrane review as gold standard. Only using the first two pages.

Table below shows the primary outcomes for search performances were sensitivity and PPV for the cut-off (the two first two pages of PubMed output). When full output was screened for relevant studies, about 85 % were detected by PIC queries and 69 % by PICO queries.

<table>
<tr><th>Strategy no</th><th>Query</th><th>Clinical Queries</th><th>Limits</th><th>Median precision</th></tr>
<tr><td>S1</td><td>PIC[d]</td><td>NA[e]</td><td>NA</td><td>2.5</td></tr>
<tr><td>S2</td><td>PIC</td><td>Therapy, broad</td><td>NA</td><td>5.0</td></tr>
<tr><td>S3</td><td>PIC</td><td>Therapy, broad</td><td>English, human</td><td>5.0</td></tr>
<tr><td>S4</td><td>PIC</td><td>Therapy, narrow</td><td>NA</td><td>21.3</td></tr>
<tr><td>S5</td><td>PIC</td><td>Therapy, narrow</td><td>English, human</td><td>23.8</td></tr>
<tr><td>S6</td><td>PICO[d]</td><td>NA</td><td>NA</td><td>6.3</td></tr>
<tr><td>S7</td><td>PICO</td><td>Therapy, broad</td><td>NA</td><td>8.8</td></tr>
<tr><td>S8</td><td>PICO</td><td>Therapy, broad</td><td>English, human</td><td>11.3</td></tr>
<tr><td>S9</td><td>PICO</td><td>Therapy, broad</td><td>English, human, AIM[f]</td><td>20.0</td></tr>
<tr><td>S10</td><td>PICO</td><td>Therapy, narrow</td><td>NA</td><td>32.1</td></tr>
<tr><td>S11</td><td>PICO</td><td>Therapy, narrow</td><td>English, human</td><td>32.8</td></tr>
<tr><td>S12</td><td>PICO</td><td>Therapy, narrow</td><td>English, human, AIM</td><td>50.0</td></tr>
<tr><td>S13</td><td>Related #1[g]</td><td>NA</td><td>NA</td><td>10.0</td></tr>
<tr><td>S14</td><td>Related #2[g]</td><td>NA</td><td>NA</td><td>10.0</td></tr>
<tr><td>S15</td><td>Related #3[g]</td><td>NA</td><td>NA</td><td>7.5</td></tr>
</table>
</td><td>pg. 8, Results

Table 2</td></tr>
</table>

JMLA Journal of the Medical Library Association

Supplemental content to
J Med Libr Assoc. Oct;106(4):dx.doi.org/10.5195/jmla.2018.345
www.jmla.mlanet.org
© Eriksen, Frandsen 2018

<table>
<tr><th></th><th>Description as stated in report/paper</th><th>Location in text
(pg. & ¶/fig/table)</th></tr>
<tr><td>Data on recall or sensitivity
(indicate how calculated)</td><td>The number of gold standard articles retrieved
<table>
<tr><th>Strategy no</th><th>Query</th><th>Clinical Queries</th><th>Limits</th><th>Median sensitivity</th></tr>
<tr><td>S1</td><td>PIC[d]</td><td>NA[e]</td><td>NA</td><td>9.8</td></tr>
<tr><td>S2</td><td>PIC</td><td>Therapy, broad</td><td>NA</td><td>14.6</td></tr>
<tr><td>S3</td><td>PIC</td><td>Therapy, broad</td><td>English, human</td><td>17.6</td></tr>
<tr><td>S4</td><td>PIC</td><td>Therapy, narrow</td><td>NA</td><td>48.5</td></tr>
<tr><td>S5</td><td>PIC</td><td>Therapy, narrow</td><td>English, human</td><td>52.8</td></tr>
<tr><td>S6</td><td>PICO[d]</td><td>NA</td><td>NA</td><td>17.9</td></tr>
<tr><td>S7</td><td>PICO</td><td>Therapy, broad</td><td>NA</td><td>26.1</td></tr>
<tr><td>S8</td><td>PICO</td><td>Therapy, broad</td><td>English, human</td><td>29.6</td></tr>
<tr><td>S9</td><td>PICO</td><td>Therapy, broad</td><td>English, human, AIM[f]</td><td>15.5</td></tr>
<tr><td>S10</td><td>PICO</td><td>Therapy, narrow</td><td>NA</td><td>54.7</td></tr>
<tr><td>S11</td><td>PICO</td><td>Therapy, narrow</td><td>English, human</td><td>54.7</td></tr>
<tr><td>S12</td><td>PICO</td><td>Therapy, narrow</td><td>English, human, AIM</td><td>15.5</td></tr>
<tr><td>S13</td><td>Related #1[g]</td><td>NA</td><td>NA</td><td>39.7</td></tr>
<tr><td>S14</td><td>Related #2[g]</td><td>NA</td><td>NA</td><td>37.9</td></tr>
<tr><td>S15</td><td>Related #3[g]</td><td>NA</td><td>NA</td><td>37.5</td></tr>
</table></td><td></td></tr>
<tr><td>Data on time spent
(if available)</td><td>N/A</td><td></td></tr>
<tr><td>Unguided search
(verbatim description)</td><td>N/A</td><td></td></tr>
<tr><td>Databases searched</td><td>PubMed</td><td></td></tr>
</table>

JMLA Journal of the Medical Library Association

SC

Supplemental content to
J Med Libr Assoc. Oct;106(4):dx.doi.org/10.5195/jmla.2018.345
www.jmla.mlanet.org
© Eriksen, Frandsen 2018

Risk of bias assessment

Criteria	Risk of bias<br>*Low/High/Unclear*	Support for judgment	Location in text<br>*(pg. & ¶/fig/table)*
Searcher skill criterion	Unclear risk of bias	Unclear searcher skill criterion. Some of the authors were probably enrolled as participants.<br>The authors planned the searches. It is unclear who performed the searches.<br>The participants (searchers) were familiar with the intervention prior to the study.	pg. 3, Methods section states that two of the authors planned the searches. Methods section as well as "Study Limitations and Strengths" indicate that the authors performed the searches. However, it is not clearly stated.
Fit between model and topic criterion	High risk of bias	The selected reviews represent "a wide spectrum of topics of general interest."<br>The topics are, however, not considered when selecting the appropriate models. PICO is probably the relevant model to include but it is unclear what the most relevant comparative model is.<br>A gold standard is used for determining relevance. However, the gold standard is used to determine relevance not for a systematic reviewer but for clinicians who "only screen the first two pages." Consequently, search strategies that produce fewer results are favored.	"Sample of Systematic Reviews for the Identification of Relevant Articles"<br>Discussion

Supplemental content to
J Med Libr Assoc. Oct;106(4):dx.doi.org/10.5195/jmla.2018.345
www.jmla.mlanet.org
© Eriksen, Frandsen 2018

Criteria	Risk of bias<br>*Low/High/Unclear*	Support for judgment	Location in text<br>*(pg. & ¶/fig/table)*
Performed searches criterion	Unclear risk of bias	Systematic reviews were used as a gold standard. Original search strategies from the included Cochrane reviews were not adapted. Search strategies included automatic Medical Subject Headings (MeSH) mapping. However, it is unclear if the automatic mapping was checked for correct translation; the query translation was not indicated for all clinical questions/searches. The search planners tried to mimic the behavior of a clinician.<br>Only PubMed was searched.	pg. 4, "Design of Search Strategies"

General information

Date form completed<br>*(dd/mm/yyyy)*	05/26/2017
Name/ID of person extracting data	Consensus extraction: Frandsen & Eriksen
Report title<br>*(title of paper/ abstract/ report that data are extracted from)*	Comparing Patient Characteristics, Type of Intervention, Control, and Outcome (PICO) Queries with Unguided Searching: A Randomized Controlled Crossover Trial
Report ID<br>*(if there are multiple reports of this study)*	Hoogendam et al.
Reference details	Hoogendam A, de Vries Robbé PF, Overbeke AJ. J Med Libr Assoc. 2012 Apr;100(2):121–6. DOI: http://dx.doi.org/10.3163/1536-5050.100.2.010.
Report author contact details	Arjen Hoogendam. Arjen.Hoogendam@gmail.com
Publication type<br>*(e.g. full report, abstract, letter)*	Full report. Journal article
Conflict of interest	Not stated.

Supplemental content to
J Med Libr Assoc. Oct;106(4):dx.doi.org/10.5195/jmla.2018.345
www.jmla.mlanet.org
© Eriksen, Frandsen 2018

Methods

**Study characteristics**	**Review inclusion criteria**<br>*All study types were included in the review*	**Yes/No/Unclear**	**Location in text**<br>*(pg. & ¶/fig/table)*
Type of study	Randomised trial	No	
	Non-randomised trial	No	
	Controlled before-after study<br>• Contemporaneous data collection<br>• At least 2 intervention and 2 control clusters	No	
	Interrupted time series OR<br>Repeated measures study<br>• At least 3 time points before and 3 after the intervention<br>• Clearly defined intervention point	No	
	Other design (specify):	Randomized controlled crossover trial	pg. 123, "Question Selection and Randomization"

JMLA Journal of the Medical Library Association

SC

Supplemental content to
J Med Libr Assoc. Oct;106(4):dx.doi.org/10.5195/jmla.2018.345
www.jmla.mlanet.org
© Eriksen, Frandsen 2018

	**Descriptions as stated in report/paper**	**Location in text** *(pg. & ¶/fig/table)*
Aim of study	The study aims to ascertain whether structuring clinical queries in the form of a PICO query, in time-restricted searches, improves search results.	pg. 121, Introduction
Participants<br>*No. and description of participants*	Of 30 invited specialists and residents from the Radboud University Nijmegen Medical Centre with interest in vascular medicine, 24 agreed to participate in the study. Eleven participants were female, 13 male; 15 were residents; 9 were specialist in internal medicine (3 fellows, 6 with a subspecialty in vascular medicine). Two physicians (1 male internist and 1 male resident) were not able to attend the second session due to causes not related to the study. Both were excluded from the analysis of results. The 22 remaining participants answered 440 questions.<br>22 participants:<br>14 residents<br>8 specialists	Results: first paragraph<br><br>pg. 123
Models included in the study<br>*Verbatim extraction*	The PICO model vs. unguided search. The participants were allowed to modify the PICOs if they wished, either by changing the content of a category or removing a category. Not all categories had to be used; for example, if no control group could be defined, it could be left out.	Methods, study protocol<br><br>pg. 122
Number and wording of clinical questions included in the study	Twenty systematic reviews dealing with vascular medicine that provided references to more than five articles available in PubMed were selected from the Cochrane database. The topics of the reviews were translated to clinical questions by the authors and checked by a librarian. The questions are available online.	Table 1

JMLA Journal of the Medical Library Association

SC

Supplemental content to
J Med Libr Assoc. Oct;106(4):dx.doi.org/10.5195/jmla.2018.345
www.jmla.mlanet.org
© Eriksen, Frandsen 2018

Outcomes

	**Description as stated in report/paper**	**Location in text** *(pg. & ¶/fig/table)*
Outcome name(s)	Recall, precision	pg. 123, Results
Data on precision *(indicate how calculated)*	Precision seemed to have been calculated by someone else than the searcher as sessions only lasted two hours and each searcher needed to search. A Cochrane review is used as gold standard.<br>Average precision: 4.02% UNGUIDED<br>Average precision: 3.44% PICO<br>Difference not significant	pg. 123, Results
Data on recall or sensitivity *(indicate how calculated)*	Recall seemed to have been calculated by someone else than the searcher as sessions only lasted two hours and each searcher needed to search. Each searcher had to hand in the best search (defined as most relevant articles), and some relevance assessment took place. A Cochrane review is used as gold standard.<br>Average recall: 12.27% UNGUIDED<br>Average recall: 13.62% PICO<br>Difference not significant.	pg. 123, Results
Data on time spent *(if available)*	N/A (Fixed time for all participants—not relevant)	
Databases searched	PubMed	pg. 122, Methods

JMLA Journal of the Medical Library Association

Supplemental content to
J Med Libr Assoc. Oct;106(4):dx.doi.org/10.5195/jmla.2018.345
www.jmla.mlanet.org
© Eriksen, Frandsen 2018

Risk of bias assessment

Criteria	Risk of bias<br>*Low/High/Unclear*	Support for judgment	Location in text<br>*(pg. & ¶/fig/table)*
Searcher skill criterion	High risk of bias	All of the participants (searchers) were invited to a one-hour PubMed lecture, but the participants (searchers) are not selected on the basis of their experience with PubMed or searching in general. Some of the participants (searchers) could have performed searches for systematic reviews. "They were all familiar with searching PubMed." Consequently, many are probably familiar with the PICO model and may thus use it unconsciously, while performing unguided searches.<br>This potential bias is not dealt with.<br>Questions are blinded but evaluators probably not.	pg. 122, Methods
Fit between model and topic criterion	Unclear risk of bias	The starting point is questions in the field of vascular medicine, and PICO is an obvious model to test. However, the unguided searches are more difficult to define as most searchers who are familiar with PubMed will use the PICO model unconsciously as it is the preferred model for training sessions.<br>It is not stated how many elements the searches consisted of; however, it is stated that the participants were allowed to remove categories (P, I, C, or O). Only PubMed was searched.	pg. 122, Study protocol<br>pg. 122–123, Study protocol (1st and last paragraph)

JMLA Journal of the Medical Library Association

SC

Supplemental content to
J Med Libr Assoc. Oct;106(4):dx.doi.org/10.5195/jmla.2018.345
www.jmla.mlanet.org
© Eriksen, Frandsen 2018

Criteria	Risk of bias<br>*Low/High/Unclear*	Support for judgment	Location in text<br>*(pg. & ¶/fig/table)*
Performed searches criterion	Unclear risk of bias	Systematic reviews were used as golden standard.<br>Searchers (participants) were allowed to use MeSH but were not allowed to use Clinical Queries or other filters. After five minutes of searching, PubMed closed automatically, and the participant was asked to record, by copying and pasting, the query that delivered the most relevant articles in the smallest set of articles.<br>Searchers (participants) received training on MeSH, details, and the like. However, it is not possible to see if they were instructed to use MeSH.<br>It is unclear if the searchers (participants) were instructed to use the automatic term mapping.	Study protocol, pg. 122

General information

Date form completed *(dd/mm/yyyy)*	05/26/2017
Name/ID of person extracting data	Consensus: Frandsen & Eriksen
Report title<br>*(title of paper/ abstract/ report that data are extracted from)*	PICO, PICOS, and SPIDER: A Comparison Study of Specificity and Sensitivity in Three Search Tools for Qualitative Systematic Reviews
Report ID<br>*(if there are multiple reports of this study)*	Methley et al.
Reference details	Methley AM, Campbell S, Chew-Graham C, McNally R, Cheraghi-Sohi S. BMC Health Serv Res. 2014 Nov 21;14:579.
Report author contact details	Abigail M Methley: abigail.methley@postgrad.manchester.ac.uk
Publication type<br>*(e.g. full report, abstract, letter)*	Full report, journal article
Conflict of interest	The authors declare that they have no competing interests.

JMLA Journal of the Medical Library Association

SC

Supplemental content to
J Med Libr Assoc. Oct;106(4):dx.doi.org/10.5195/jmla.2018.345
www.jmla.mlanet.org
© Eriksen, Frandsen 2018

Methods

**Study characteristics**	**Review inclusion criteria**<br>*All study types were included in the review*	**Yes/No/Unclear**	**Location in text**<br>*(pg. & ¶/fig/table)*
Type of study	Randomised trial	No	
	Non-randomised trial	No	
	Controlled before-after study<br>• Contemporaneous data collection<br>• At least 2 intervention and 2 control clusters	No	
	Interrupted time series OR<br>Repeated measures study<br>• At least 3 timepoints before and 3 after the intervention<br>• Clearly defined intervention point	No	
	Other design (specify):	Observational study	The study design is not stated in the paper.

JMLA Journal of the Medical Library Association

SC

Supplemental content to
J Med Libr Assoc. Oct;106(4):dx.doi.org/10.5195/jmla.2018.345
www.jmla.mlanet.org
© Eriksen, Frandsen 2018

	**Descriptions as stated in report/paper**	**Location in text** *(pg. & ¶/fig/table)*
Aim of study	The aim of this article was to test SPIDER by broadly replicating the work of Cooke et al. [9], specifically by comparing the two approaches: (1) the traditional PICO method of searching electronic databases with (2) the newly devised SPIDER tool, developed for qualitative and mixed-method research. In addition, we wished to build and expand on the work of Cooke et al. [9] and so our third aim was to compare PICO and SPIDER to a modified PICO with qualitative study designs<br>(PICOS, see Table 1 by investigating specificity and sensitivity across 3 major databases.)	pg. 3, last paragraph
Participants<br>*No and description of participants*	A search strategy was developed as collaboration between some or all of the authors of the paper and a specialist librarian and information specialist.	pg. 4, "Search Strategy," 1st paragraph
Models included in the study<br>*Verbatim extraction*	SPIDER, PICO, and PICOS	pg. 4, "Search Strategy," 1st paragraph
Number and wording of clinical questions included in the study	One clinical question used as basis for the searches, regardless of the model used.<br>Studies eligible for inclusion were those that qualitatively investigated patients' experiences, views, attitudes to, and perceptions of health care services for multiple sclerosis.	pg. 4., Methods, "Inclusion and Exclusion Criteria" (1st paragraph)

JMLA Journal of the Medical Library Association

SC

Supplemental content to
J Med Libr Assoc. Oct;106(4):dx.doi.org/10.5195/jmla.2018.345
www.jmla.mlanet.org
© Eriksen, Frandsen 2018

Outcomes

	**Description as stated in report/paper**	**Location in text (*pg. & ¶/fig/table*)**
Outcome name(s)	Sensitivity defined as % relevant texts identified out of all relevant hits.<br>Specificity defined as % relevant texts identified out of all hits.<br>"*All three databases were checked for all articles*"; however, it is unclear which of the so called relevant articles are actually indexed in the databases searched.	Table 7<br>pg. 9, last paragraph above Discussion
Outcome definition of relevance	The authors of the paper judge the relevance of a specific paper. Their background for this work is not stated.	
Data on precision<br>*(indicate how calculated)*	Search tool and database — Sensitivity (%)<br>CINAHL PICO — 14/1,350=1.04<br>CINAHL PICO S — 12/146=8.22<br>CINAHL SPIDER — 12/146=8.22<br>MEDLINE PICO — 12/8,158=0.15<br>MEDLINE PICO S — 6/113=5.32<br>MEDLINE SPIDER — 5/14=35.71<br>EMBASE PICO — 14/14,250=0.1<br>EMBASE PICO S — 7/189=3.7<br>EMBASE SPIDER — 3/55=5.45	Table 7

JMLA Journal of the Medical Library Association

SC

Supplemental content to
J Med Libr Assoc. Oct;106(4):dx.doi.org/10.5195/jmla.2018.345
www.jmla.mlanet.org
© Eriksen, Frandsen 2018

	**Description as stated in report/paper**	**Location in text** ***(pg. & ¶/fig/table)***
Data on recall or sensitivity *(indicate how calculated)*	They do not use a systematic review as a gold standard. Recall is calculated in each database using the total number of relevant papers as gold standard. They do not adjust for actual indexing. Search tool and database — Sensitivity (%) CINAHL PICO — 14/18=77.78 CINAHL PICO S — 12/18=66.67 CINAHL SPIDER — 12/18=66.67 MEDLINE PICO — 12/18=66.67 MEDLINE PICO S — 6/18=33.33 MEDLINE SPIDER — 5/18=27.78 EMBASE PICO — 13/18=72.22 EMBASE PICO S — 7/18=38.88 EMBASE SPIDER — 3/18=16.67	Table 7
Data on time spent *(if available)*	N/A—Not stated	
Databases searched	CINAHL, Embase, MEDLINE	pg. 4, Methods, "Search Strategy"

JMLA Journal of the Medical Library Association

SC

Supplemental content to
J Med Libr Assoc. Oct;106(4):dx.doi.org/10.5195/jmla.2018.345
www.jmla.mlanet.org
© Eriksen, Frandsen 2018

Risk of bias assessment

**Criteria**	**Risk of bias** *Low/High/Unclear*	**Support for judgment**	**Location in text** *(pg. & ¶/fig/table)*
Searcher skill criterion	Unclear risk of bias	The article states that "we developed a detailed search strategy in collaboration with a specialist librarian and information specialist." The skills level of each participant (searcher) is unclear both medical as well as searching.<br><br>It is unclear who exactly participated and if all of the participants (searchers) received all interventions, if the participants (searchers) were familiar with the interventions prior to the study.<br><br>There was no blinding it seems, and the searcher is unclear.	pg. 4, Methods, "Search Strategy"
Fit between model and topic criterion	High risk of bias	A qualitative question is selected to test several models, but they are developed to fit different topics and apply to some topics better than others. The topic is not taken into consideration in relation to the intervention (to fit the models).<br><br>The number of search blocks varies from 4 to 6 and consequently affects recall as well as precision. This is not taken into consideration in the discussion of the findings.	pg. 4, Methods, "Inclusion and Exclusion Criteria"<br><br>Table 2, 3, and 4
Performed searches criterion	Low risk of bias	An already existing systematic review was not used as a gold standard as evaluation of the effect of the interventions.<br><br>The literature searches were performed adequately according to the database as regards the use of subject terms and text words. Several databases were used, and there seems to be a slight lack of consensus between the searches in the different databases for some text words (e.g., "consumer" was searched as a text word in Embase and MEDLINE, but not in CINAHL Plus).	Table 2, 3, and 4

JMLA Journal of the Medical Library Association

SC

Supplemental content to
J Med Libr Assoc. Oct;106(4):dx.doi.org/10.5195/jmla.2018.345
www.jmla.mlanet.org
© Eriksen, Frandsen 2018

Reference

1. Higgins JPT, Green S, eds. Cochrane handbook for systematic reviews of interventions. Chichester, UK: John Wiley & Sons; 2008. Higgins JPT, Deeks JJ, eds. Chapter 7, Selecting studies and collecting data.
